# miR-21-5p attenuates hyperoxia-induced lung injury by modulating YAP1-dependent ferroptosis

**DOI:** 10.3389/fphar.2026.1804152

**Published:** 2026-05-11

**Authors:** Qianxia Huang, Ping Yuan, GuoYue Liu, Guiyang Jia, Erqin Song, Kangjie Qin, Zhihui Wang, Fengmin Yin, Miao Chen

**Affiliations:** 1 Department of Critical Care Medicine, The Third Affiliated Hospital of Zunyi Medical University (The First People’s Hospital of Zunyi), Zunyi, China; 2 Affiliated Hospital of Zunyi Medical University, Zunyi, China; 3 Children’s Hospital of Guizhou Province, Zunyi, China; 4 Department of Critical Care Medicine, The Second Affiliated Hospital of Zunyi Medical University, Zunyi, China; 5 Department of Critical Care Medicine, Kweichow Moutai Hospital, Zunyi, China

**Keywords:** ferroptosis, hyperoxia-induced acute lung injury, miR-21-5p, oxidative stress, YAP1

## Abstract

**Background:**

Hyperoxia-induced acute lung injury (HALI) is a frequent and clinically relevant complication of oxygen therapy in critically ill patients. Excessive oxygen exposure induces severe oxidative stress and alveolar epithelial cell (AEC) injury, yet the upstream regulatory mechanisms governing regulated cell death under hyperoxic conditions remain incompletely understood. Ferroptosis, an iron-dependent form of lipid peroxidation–driven cell death, has recently been implicated in HALI pathogenesis.

**Methods:**

In this study, hyperoxia-induced AEC injury models *in vitro* and a murine HALI model *in vivo* were established to investigate the role of miR-21-5p in ferroptosis regulation. Gain- and loss-of-function approaches were combined with molecular, biochemical, and histological analyses to evaluate ferroptosis-related phenotypes. The regulatory interaction between miR-21-5p and Yes-associated protein 1 (YAP1) was examined using dual-luciferase reporter and RNA immunoprecipitation assays.

**Results:**

Hyperoxic exposure markedly induced ferroptosis-associated features in AECs and lung tissue, characterized by increased lipid peroxidation, iron accumulation, and impaired antioxidant capacity. miR-21-5p expression was significantly downregulated under hyperoxic conditions, whereas YAP1 expression was increased. miR-21-5p directly targeted YAP1 and negatively regulated its expression. Restoration of miR-21-5p attenuated hyperoxia-induced ferroptosis, reduced oxidative stress, and improved lung injury, while miR-21-5p deficiency exacerbated ferroptosis-related alterations and aggravated HALI. Mechanistically, modulation of the miR-21-5p/YAP1 axis was associated with coordinated changes in key ferroptosis-related molecules, including ACSL4, SLC7A11, and GPX4.

**Conclusion:**

These findings identify miR-21-5p as an important upstream regulator of ferroptosis in hyperoxia-induced lung injury through YAP1-dependent mechanisms. The miR-21-5p/YAP1 axis contributes to redox imbalance and ferroptotic susceptibility in HALI, highlighting a potential regulatory pathway relevant to hyperoxia-associated pulmonary injury.

## 1 Introduction

Oxygen therapy is indispensable for maintaining adequate tissue oxygenation in critically ill patients. However, accumulating evidence indicates that excessive oxygen administration is associated with substantial pulmonary toxicity. Exposure to high oxygen concentrations disrupts the alveolar–capillary barrier and can lead to hyperoxia-induced acute lung injury (HALI), which may progress to acute respiratory distress syndrome (ARDS) or neonatal bronchopulmonary dysplasia (BPD) (Cox et al., 2015; Syed et al., 2017). Epidemiological studies report mortality rates ranging from 35% to 45% in patients with HALI and related complications (Bellani et al., 2016). Despite advances in critical care, hyperoxia-associated lung injury remains prevalent during clinical interventions such as mechanical ventilation, cardiopulmonary resuscitation, and neonatal resuscitation, representing a persistent clinical challenge worldwide (Damiani et al., 2014; Girardis et al., 2016).

Hyperoxic lung injury is largely driven by excessive production of reactive oxygen species (ROS). Elevated ROS levels promote lipid peroxidation, inflammatory signaling, and multiple forms of regulated cell death, thereby compromising the alveolar–capillary barrier, impairing gas exchange, and inducing alveolar edema (Singer et al., 2021). ROS directly damage alveolar epithelial and capillary endothelial cells and oxidize lipids, proteins, and DNA, creating a self-amplifying cycle of oxidative stress, membrane injury, and inflammatory activation that contributes to ARDS progression (Laube et al., 2017; Liu et al., 2025; Zhou et al., 2025). In addition to oxidative stress and apoptosis, mitochondrial dysfunction, endoplasmic reticulum stress, and dysregulated autophagy have also been implicated in HALI pathogenesis (Sureshbabu et al., 2016; Zhu et al., 2024).

Recently, ferroptosis—an iron-dependent form of regulated cell death driven by lipid peroxidation—has attracted increasing attention in the context of oxidative stress–related lung injury (Sun et al., 2025). First described by Dixon et al., in 2012, ferroptosis is mechanistically and morphologically distinct from apoptosis, necrosis, pyroptosis, and autophagic cell death. Ferroptotic cells are characterized by mitochondrial shrinkage, loss of cristae, increased membrane density, and preserved nuclear integrity. At the biochemical level, ferroptosis is associated with iron accumulation, excessive lipid peroxide generation, glutathione (GSH) depletion, and reduced activity of glutathione peroxidase 4 (GPX4) (Tang et al., 2021; Stockwell, 2022). At the molecular level, acyl-CoA synthetase long-chain family member 4 (ACSL4) promotes the incorporation of polyunsaturated fatty acids into membrane phospholipids, thereby increasing susceptibility to lipid peroxidation (Lee and Gan, 2022). In contrast, the cystine/glutamate antiporter subunit solute carrier family 7 member 11 (SLC7A11, also known as xCT), together with GPX4, supports cystine uptake, glutathione synthesis, and phospholipid hydroperoxide detoxification, serving as central components of cellular redox and antioxidant homeostasis (Dasgupta and Gan, 2022).

Within the ferroptosis regulatory network, microRNAs (miRNAs) have emerged as important post-transcriptional modulators (Guo B. et al., 2022). miRNAs are small non-coding RNAs (∼22–24 nucleotides) that regulate diverse cellular processes, including proliferation, differentiation, and cell death, by binding to target mRNAs to suppress translation or promote degradation (Ferruelo et al., 2018; Kilikevicius et al., 2022; Lu et al., 2022; Wang et al., 2025). Among these, miR-21-5p—a key member of the miR-21 family—has been implicated in multiple pulmonary disorders, such as lung cancer, asthma, ARDS, and chronic obstructive pulmonary disease (COPD) (Ren et al., 2019; Ding et al., 2021; Mao et al., 2024). Previous studies have demonstrated that hyperoxic exposure is associated with significant downregulation of miR-21-5p in alveolar type II epithelial cells (AEC II) (He et al., 2017). This reduction has been linked to increased HALI severity through modulation of autophagy and apoptosis via the PTEN/Akt pathway (Qin et al., 2019). In addition, miR-21-5p inhibits PGAM5-mediated necroptosis through exosome-dependent mechanisms (Yuan et al., 2025), whereas the transcription factor Gata3 enhances miR-21-5p transcription to attenuate AEC apoptosis and lung injury (Ren et al., 2025). Collectively, these findings suggest that miR-21-5p functions as a cytoprotective regulator under hyperoxic stress.

Yes-associated protein 1 (YAP1), a core transcriptional co-activator of the Hippo signaling pathway, plays a key role in regulating cell proliferation, differentiation, and stress-responsive signaling (Ishihara and Nishina, 2018). Dysregulated YAP1 activity has been implicated in a wide range of pathological conditions, including solid tumors, cardiovascular diseases, and pulmonary fibrosis (Fodor et al., 2017). Notably, in sepsis-induced acute lung injury, YAP1 deficiency has been reported to reduce GPX4 and SLC7A11 expression while increasing nuclear receptor coactivator 4 (NCOA4), thereby enhancing ferroptosis susceptibility (Zhang et al., 2022). In parallel, miR-21-5p–enriched mesenchymal stem cell–derived exosomes have been shown to attenuate myocardial ischemia–reperfusion injury through suppression of YAP1 expression (Ji and Wang, 2024). However, the role of YAP1 in hyperoxia-induced lung injury and its potential regulation by miR-21-5p remain poorly defined.

Our preliminary microarray analysis revealed significant downregulation of miR-21-5p in hyperoxia-exposed AEC II (Qin et al., 2018). However, whether miR-21-5p regulates ferroptosis through YAP1 signaling in the context of hyperoxia-induced lung injury remains unclear.In this study, we examined the role of the miR-21-5p/YAP1 axis in HALI using complementary *in vitro* gain- and loss-of-function approaches and an *in vivo* miR-21-5p knockout mouse model, with a focus on ferroptosis-associated processes and related molecular changes.

## 2 Materials and Methods

### 2.1 Bioinformatics analysis methods

To identify candidate genes associated with hyperoxic lung injury, datasets were retrieved from the Gene Expression Omnibus (GEO) database using the keywords “hyperoxia” and “lung,” with the species restricted to mice. Based on data completeness and relevance, three datasets (GSE51039, GSE25293, and GSE58654) were selected for further analysis. Differential expression analysis was performed for each dataset using the GEO2R online tool, with the normoxic group as the control and the hyperoxic group as the experimental condition. Differentially expressed genes (DEGs) were identified using thresholds of adjusted *p*-value <0.05 and |log2 fold change| > 2.0. The results were visualized as volcano plots and heatmaps generated by GEO2R. The identified DEGs were subjected to functional enrichment analysis. Gene Ontology (GO) annotation and Kyoto Encyclopedia of Genes and Genomes (KEGG) pathway enrichment analyses were conducted using Metascape (https://metascape.org) with default parameters (minimum overlap = 3, *p*-value cutoff = 0.01). Enrichment results were visualized using the online platform Weishengxin (https://www.bioinformatics.com.cn).

Potential target genes of miR-21-5p were predicted using TargetScan (https://www.targetscan.org/vert_80/), starBase (https://rnasysu.com/encori/), TarBase (https://dianalab.e-ce.uth.gr/tarbasev9), and miRDB (https://mirdb.org/), while ferroptosis-related genes were obtained from the FerrDb database (http://www.zhounan.org/ferrdb/current/). Subsequently, an intersection analysis was performed among predicted miR-21-5p targets, ferroptosis-related genes, and DEGs, and the overlapping genes were visualized using Venn diagrams generated via the Weishengxin platform. Based on literature evidence and expression patterns, YAP1 was selected for subsequent experimental validation.

### 2.2 Cell culture, processing, and transfection

In this study, the mouse alveolar epithelial cell line TC-1 (Shanghai Fuheng Biotechnology Co., Ltd.) was cultured under standard conditions (37 °C, 5% CO_2_, and 21% O_2_) in RPMI 1640 medium (Gibco, 12633020) supplemented with 10% fetal bovine serum and 1% penicillin–streptomycin. To simulate hyperoxic conditions, cells were incubated in a hyperoxic culture chamber (Heal Force) at 37 °C with 85% O_2_ for 36 h. In selected experiments, the ferroptosis inhibitor Ferrostatin-1 (Fer-1; MCE, HY-100579) was added to the culture medium at a final concentration of 1 μM during hyperoxic exposure. The control group received an equal volume of dimethyl sulfoxide (DMSO, Solarbio, P6110M) as a vehicle control.

Cell transfection was performed using Opti-MEM™ (Thermo Fisher Scientific, 31985070) and Lipofectamine 3000 (Thermo Fisher Scientific, L3000015). Transfections were carried out in 6-cm culture dishes. miR-21-5p mimics/inhibitors, siYAP1, and their respective negative controls were transfected at a final amount of 150 pmol per dish (stock concentration: 20 pmol/μL). The YAP1 overexpression plasmid and corresponding empty vector were transfected at 2.5 μg per dish (plasmid concentration: 500 ng/μL). Cells were harvested 48 h post-transfection for RNA and protein extraction.

The sequences of the miR-21-5p mimic, inhibitor, negative control, and YAP1-targeting siRNA are listed in Supplementary Table S1. All sequences were designed by Shanghai Jima Pharmaceutical Technology Co., Ltd. The YAP1 overexpression vector was generated by cloning the full-length mouse YAP1 coding sequence into the pEX-3 vector, with the empty vector used as the plasmid control. Mimic NC and inhibitor NC were non-specific oligonucleotides lacking known target specificity, siNC was a scrambled siRNA, and OE NC consisted of the same vector backbone without the YAP1 insert.

### 2.3 Animals and hyperoxic lung injury model

Six-to eight-week-old C57BL/6 wild-type (WT) mice were purchased from Hunan Slack Jingda Laboratory Animal Co., Ltd. (license No. SCXK [Xiang] 2021-0002), and miR-21-5p knockout (KO) mice were generated by Cyagen Biosciences (license No. SCXK [Su] 2022-0016). Mice were randomly assigned to control, hyperoxia, and treatment groups, with six mice per group.Hyperoxia exposure was achieved by placing mice in a custom-designed oxygen chamber (Puhe Biotechnology) and maintaining them in 85% O_2_ for 72 h to establish a hyperoxia-induced lung injury model. In the Fer-1 treatment group, mice received an intraperitoneal injection of Ferrostatin-1 (5 mg/kg, dissolved in 0.1% DMSO and diluted with 0.9% NaCl) 2 h prior to hyperoxia exposure, followed by continuous exposure for 72 h.

At the end of exposure, mice were euthanized under anesthesia with tribromoethanol (800 μL, intraperitoneal injection), and lung tissues and bronchoalveolar lavage fluid (BALF) were collected for subsequent analyses. All procedures were approved by the Animal Ethics Committee of Zunyi Medical University (Approval No. ZMU21-2411-003) and conducted in accordance with national laboratory animal regulations and ARRIVE guidelines (https://arriveguidelines.org).

### 2.4 Cell viability assay using Cell Counting Kit-8 (CCK-8)

TC-1 cells were seeded in 96-well plates at a density of 6–8 × 10^3^ cells per well in 100 µL complete medium and incubated for 24 h. Following treatment and hyperoxia exposure, 10 µL of CCK-8 (Uelandy, C6005M)reagent was added to each well under dark conditions. Plates were incubated at 37 °C for 2 h, and absorbance was measured at 450 nm using a microplate reader. Cell viability was calculated and subjected to statistical analysis.

### 2.5 Reverse transcription and quantitative PCR (RT-qPCR)

After treatment, the culture medium was discarded, cells were gently washed with phosphate-buffered saline (PBS, Solarbio, P1010), and adherent cells were collected for RNA extraction. Total RNA was extracted using TRIzol reagent (Takara, 9108). RT-qPCR were performed using SYBR Green Premix (Servicebio, G3326-01) with gene-specific primers. The cycling conditions were as follows: pre-denaturation at 95 °C for 30 s, followed by 40 cycles of 95 °C for 15 s, 65 °C for 10 s, and 72 °C for 30 s. Relative gene expression was calculated using the 2^−ΔΔCt^ method. U6 served as the internal control for miRNA quantification, and β-actin served as the reference gene for mRNA. Primers were synthesized by Qingke Biotechnology, and sequences are listed in Table 1.

**TABLE 1:** RT-qPCR Primer sequence.

Name	Sequences(5′-3′)
miR-21-5p	RT GTCGTATCCAGTGCAGGGTCCGAGGTATTC F AAGAGCGTTAGCTTATCAGACTG R CAGTGCAGGGTCCGAGGT
U6	RT GGAACGCTTCACGAATTTG F ATTGGAACGATACAGAGAAGATT R GGAACGCTTCACGAATTTG
YAP1	F GTCACGCACGATTTCCCTCTCAG R TATGCTCTCCCTCACGCCATCC
ACSL4	F TGCAGCAGTTACAGATGGAGAAG R TTGCTGTGCTGGGATTGATATTC
SLC7A11	F ATTGTGATCGGTGGCCAGAATAT R GACAATTCTTCAGTGCAGCTTCT
GPX4	F TCTTCGATACAAACGCCCAGATA R GTGCTGAATGGGTCCGAGTAAAG
β-actin	F CAAGTTTGACATGTACAGCAAGATCT R CACGGCAGGTCCTTCTCTATCA

### 2.6 Western blot

After treatment, the culture medium was discarded, cells were gently washed with PBS, and adherent cells were collected for protein extraction. Tissues or cells were lysed in RIPA buffer (200 µL per 20 mg tissue or cell pellet), centrifuged at 12,000 × g for 15 min at 4 °C, and supernatants were collected. Protein concentration was determined using a BCA assay. Samples were denatured at 100 °C for 7 min, separated by SDS-PAGE (20–30 µg per lane; 220 V, 45 min), and transferred to PVDF membranes (400 mA, 30 min). Membranes were blocked and incubated with primary antibodies overnight at 4 °C, followed by HRP-conjugated secondary antibodies for 1 h at room temperature. Signals were visualized using ECL reagents and quantified with ImageJ. Primary antibodies included anti-YAP1 (CST, #14074, 1:1000), anti-ACSL4 (HUABIO, ET7111-43, 1:2000), anti-SLC7A11 (HUABIO, HA721868, 1:1000), anti-GPX4 (HUABIO, ET1706-45, 1:2000), anti-Ago2 (HUABIO, ET1702-39, 1:2000), and anti-β-actin (HUABIO, M1210-2, 1:20000). Secondary antibodies included HRP-labeled goat anti-mouse IgG (HUABIO, HA1006, 1:50000) and goat anti-rabbit IgG (HUABIO, HA1001, 1:50000).

### 2.7 Dual-luciferase reporter assay

293T cells were seeded in 24-well plates at a density of 5 × 10^4^ cells/well and cultured for 24 h before transfection. Cells were co-transfected with empty, wild-type (WT), or mutant (Mut) reporter vectors along with corresponding miRNA mimics or negative controls using Lipofectamine 3000. After 48 h, cells were lysed, and luciferase activity was assessed using a Dual-Luciferase Reporter Assay Kit (UE, F6075M). Firefly luciferase activity was normalized to Renilla luciferase activity to determine relative expression.

### 2.8 RNA immunoprecipitation (RIP)

Approximately 2 × 10^7^ cells were collected, lysed, and treated with DNase. The lysate was divided into IP, IgG control, and Input fractions. Each fraction was incubated overnight at 4 °C with target or isotype control antibodies, followed by incubation with pre-washed protein A/G magnetic beads. After washing, RNA was eluted, extracted using TRIzol, purified, dissolved in RNase-free water, and subjected to RT-qPCR analysis to assess RNA enrichment.

### 2.9 Hematoxylin–eosin (HE) staining

Paraffin-embedded lung tissues were sectioned at 5 μm thickness. The sections were then dewaxed, rehydrated, and stained with hematoxylin (Solarbio, G1140) for 3–5 min. Sections were differentiated with 1% acid alcohol for 5 s, blued with 1% ammonia for 1 min, and counterstained with eosin (Solarbio, G1100) for 1–3 min. After dehydration and xylene clearing, slides were mounted with neutral resin. Slides were observed under an optical microscope (Olympus CKX53, Olympus, Japan) and imaged for analysis (scale bar = 50 μm and 100 μm). Lung injury was scored based on neutrophil infiltration in alveolar and interstitial spaces, hyaline membrane formation, proteinaceous debris, and septal thickening (Kulkarni et al., 2022).

### 2.10 Immunofluorescence staining

Paraffin-embedded sections were deparaffinized and rehydrated, followed by antigen retrieval using Tris-EDTA buffer for approximately 30 min. After cooling naturally to room temperature, the sections were rinsed with PBS. The sections were then blocked with 5% goat serum at room temperature for 20 min. Subsequently, the sections were incubated separately with primary antibodies against ACSL4 (Proteintech, 66617-1-Ig, 1:200) or GPX4 (Proteintech, 67763-1-Ig, 1:200) overnight at 4 °C in a humidified chamber. After washing with PBS the following day, the sections were incubated with Alexa Fluor 594-conjugated goat anti-mouse IgG secondary antibody (Servicebio, GB28303, 1:500) at 37 °C for 1 h in a humidified chamber. The sections were thoroughly washed with PBS between each step.Finally, the sections were mounted with an anti-fade mounting medium containing DAPI and observed under a fluorescence microscope (Leica DMi8, Leica Microsystems, Germany) (scale bar = 100 μm).

### 2.11 Prussian blue staining

Paraffin sections were dewaxed in xylene, rehydrated through graded ethanol, and rinsed with water. Sections were incubated in Prussian blue staining solution for 1 h, washed, and counterstained with nuclear red for 1–5 min. After mounting, the slides were observed under an optical microscope (Olympus CKX53, Olympus, Japan) and imaged for analysis (scale bar = 50 μm).

### 2.12 Statistical analysis

All experiments were performed with six independent biological replicates. Data are presented as mean ± standard deviation (mean ± SD). Prior to statistical analysis, data distribution was assessed using the Shapiro–Wilk test for normality, and variance homogeneity was evaluated using the Brown–Forsythe test. For datasets that were normally distributed with equal variances, comparisons between two groups were performed using independent-samples t tests, and comparisons among multiple groups were conducted using one-way analysis of variance (ANOVA) followed by Tukey’s *post hoc* test. For datasets that were normally distributed but exhibited unequal variances, multiple-group comparisons were analyzed using Welch’s ANOVA with Dunnett’s T3 *post hoc* test. For data that did not follow a normal distribution, non-parametric tests were applied. Comparisons between two groups were performed using the Mann–Whitney U test, while comparisons among multiple groups were conducted using the Kruskal–Wallis test. Image quantification was performed using ImageJ software (version 1.8.0), and statistical analyses were conducted with GraphPad Prism software (version 10.1). For each sample, at least five randomly selected fields of view were analyzed, and quantification was performed by two independent researchers under blinded conditions. A p-value <0.05 was considered statistically significant.

## 3 Results

### 3.1 Prediction of miR-21-5p downstream targets and Association with ferroptosis

**FIGURE 1:**
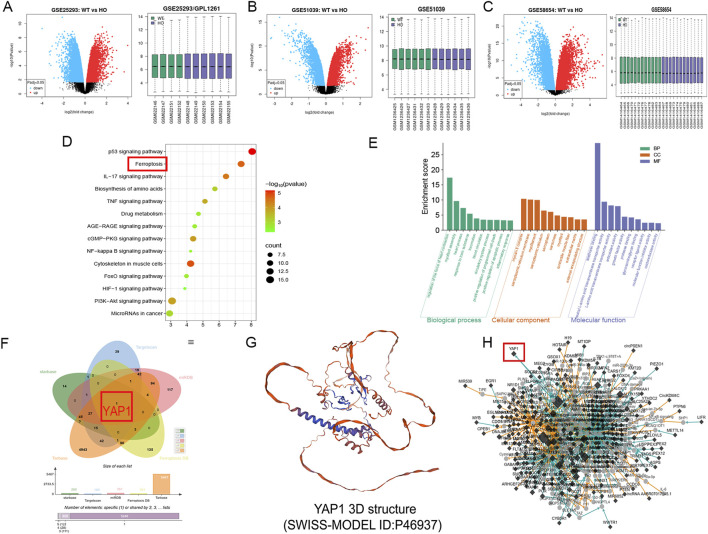
Identification of differentially expressed genes (DEGs) and prediction of miR-21-5p target genes in hyperoxia-induced lung injury **(A)** GSE25293 volcano plot and box plot. **(B)** GSE51039 volcano plot and box plot. **(C)** GSE58654 volcano plot and box plot. **(D)** Kyoto Encyclopedia of Genes and Genomes (KEGG) pathway enrichment analysis of DEGs. **(E)** Gene Ontology (GO) functional enrichment analysis of DEGs. **(F)** Venn diagram showing the intersection of predicted miR-21-5p target genes, ferroptosis-related genes, and hyperoxia-associated DEGs. **(G)** Three-dimensional structure model of Yes-associated protein 1 (YAP1). **(H)** Regulatory network of YAP1-associated molecules obtained from the FerrDb database. Diamond-shaped nodes represent protein-coding genes, while circular nodes indicate non-coding RNAs, including miRNAs, lncRNAs, and circRNAs. Node color reflects annotation category, with black nodes representing ferroptosis-related genes curated in FerrDb and gray nodes indicating associated or predicted regulatory molecules. Node size corresponds to the degree of connectivity within the network.

To explore the potential mechanisms by which miR-21-5p participates in hyperoxia-induced pulmonary injury, three public transcriptomic datasets (GSE25293, GSE51039, and GSE58654) derived from mouse HALI models were systematically analyzed. Volcano plots were used to identify DEGs, while box plots were generated to assess the distribution and normalization of gene expression values across samples, indicating comparable data quality among groups (Figures 1A–C). Functional enrichment analysis revealed that these DEGs were primarily associated with p53 signaling, ferroptosis, and inflammatory responses (Figures 1D,E).

Potential miR-21-5p target genes were predicted using TargetScan, starBase, TarBase, and miRDB databases. By intersecting the predicted miR-21-5p target genes, FerrDb ferroptosis-related genes, and hyperoxia-associated differentially expressed genes, YAP1 was retained as a candidate gene for subsequent validation. (Figures 1F,G). As a core effector of the Hippo pathway, YAP1 is known to regulate cell proliferation, apoptosis, and stress-responsive signaling. The Ferroptosis Database further annotates YAP1 as a gene potentially involved in ferroptosis regulation (Figure 1H).

### 3.2 *In Vitro* validation of direct interaction between miR-21-5p and YAP1

**FIGURE 2:**
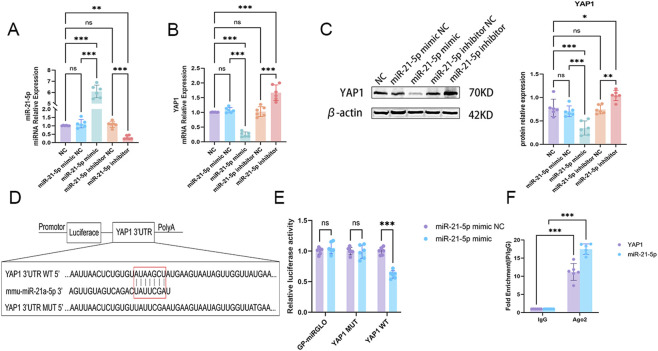
miR-21-5p directly targets and suppresses YAP1 expression in alveolar epithelial cells (AECs) **(A)** Relative expression of miR-21-5p in cells transfected with miR-21-5p mimic, inhibitor, or corresponding negative controls (NC), as determined by quantitative real-time polymerase chain reaction (RT-qPCR). **(B)** Relative mRNA expression of YAP1 following miR-21-5p modulation, measured by RT-qPCR. **(C)** Western blot analysis and quantification of YAP1 protein expression following miR-21-5p modulation. **(D)** Schematic representation of the predicted binding site between miR-21-5p and the 3′untranslated region (3′UTR) of YAP1 mRNA, including wild-type (WT) and mutant (MUT) constructs. **(E)** Dual-luciferase reporter assay showing the interaction between miR-21-5p and YAP1 3′UTR. **(F)** RNA immunoprecipitation (RIP) assay was performed using an anti-Ago2 antibody, with IgG as a negative control, to validate the interaction between miR-21-5p and YAP1. Data are presented as mean ± SD from six independent biological replicates (n = 6). Statistical analysis was performed as described in the Materials and Methods. *p < 0.05, **p < 0.01, ***p < 0.001.

Based on bioinformatics predictions, we investigated *in vitro* whether miR-21-5p targets YAP1 and modulates ferroptosis in alveolar epithelial cells. Gain- and loss-of-function experiments were performed to modulate miR-21-5p and YAP1 expression in AECs. RT-qPCR analysis confirmed that the miR-21-5p mimic significantly increased miR-21-5p expression, whereas the inhibitor reduced its expression, indicating effective transfection (Figure 2A). miR-21-5p overexpression significantly decreased YAP1 mRNA and protein levels, whereas miR-21-5p inhibition increased YAP1 expression (Figures 2B,C), suggesting a negative regulatory relationship between miR-21-5p and YAP1.

Bioinformatic analysis identified putative miR-21-5p binding sites within the YAP1 3′untranslated region (3′UTR) (Figure 2D). Dual-luciferase reporter assays showed that miR-21-5p significantly decreased luciferase activity of the wild-type YAP1 3′UTR reporter, but not that of the mutant construct (Figure 2E). RNA immunoprecipitation assays further showed co-enrichment of miR-21-5p and YAP1 mRNA within the Ago2-containing RNA-induced silencing complex (Figure 2F), supporting a potential direct post-transcriptional interaction.

### 3.3 Hyperoxic exposure is associated with ferroptosis features in AECs

**FIGURE 3:**
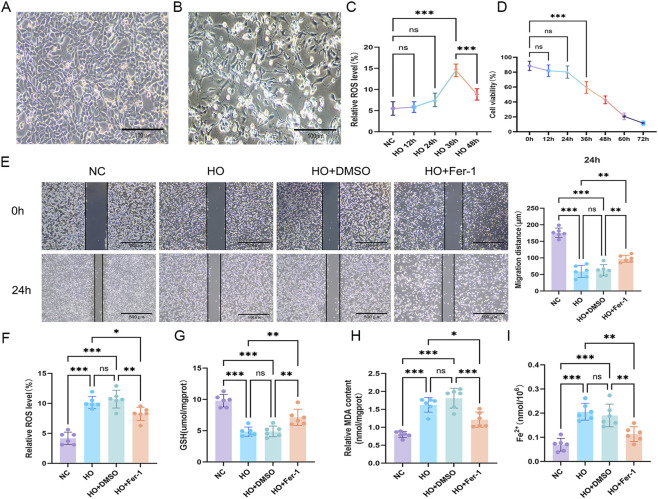
Hyperoxia induces ferroptosis in AECs, and ferrostatin-1 (Fer-1) exerts protective effects **(A)** Morphological features of TC-1 cells under normoxic conditions (control group). **(B)** Morphological changes of TC-1 cells following 36 h of hyperoxia exposure (hyperoxia group) (scale bar = 100 μm). **(C)** Intracellular reactive oxygen species (ROS) levels at different time points following hyperoxia exposure. **(D)** Cell viability measured by Cell Counting Kit-8 (CCK-8) assay at different time points. **(E)** Cell migration assessed under normoxia, hyperoxia, and hyperoxia + Fer-1 treatment (scale bar = 500 μm). **(F–I)** Levels of ROS, glutathione (GSH), malondialdehyde (MDA), and Fe^2+^. Data are presented as mean ± SD from six independent biological replicates (n = 6). Statistical analysis was performed as described in the Materials and Methods. *p < 0.05, **p < 0.01, ***p < 0.001.

Following confirmation that miR-21-5p directly targets YAP1, we next assessed whether hyperoxic exposure induces ferroptosis-related changes in AECs. Under normoxic conditions, TC-1 cells exhibited intact morphology and stable adhesion. After 36 h exposure to 85% O_2_, cells displayed reduced volume, irregular morphology, and decreased adhesion (Figures 3A,B). Reactive oxygen species (ROS) detection and CCK-8 assays revealed increased intracellular ROS levels and reduced cell viability, with maximal effects observed at 36 h of hyperoxia (Figures 3C,D). Based on these observations, 36 h was selected as the experimental time point for subsequent *in vitro* analyses.

Treatment with the ferroptosis inhibitor Fer-1 partially improved cell migratory capacity (Figure 3E) and reduced ROS, malondialdehyde (MDA), and Fe^2+^ levels while restoring GSH content (Figures 3F–I). Given that MDA reflects lipid peroxidation, Fe^2+^ contributes to ROS generation via the Fenton reaction, and GSH functions as a key antioxidant (Stockwell et al., 2017; Li et al., 2020), these results indicate that hyperoxic exposure is associated with ferroptosis-related biochemical alterations that are sensitive to Fer-1 treatment.

### 3.4 Hyperoxia activated ferroptosis pathway accompanied by downregulation of miR-21-5p and upregulation of YAP1

**FIGURE 4:**
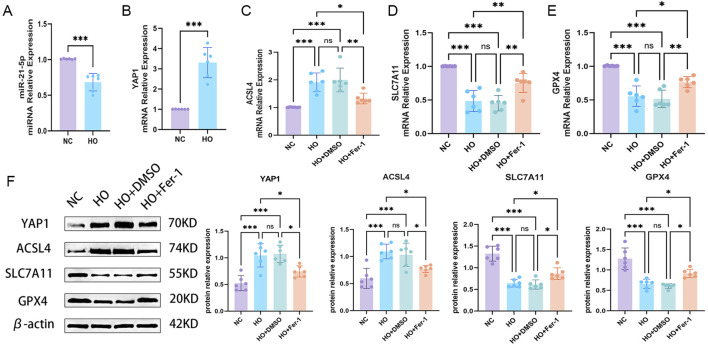
Hyperoxia is associated with ferroptosis-related changes accompanied by downregulation of miR-21-5p and upregulation of YAP1 **(A,B)** RT-qPCR analysis of miR-21-5p and YAP1 expression following exposure to hyperoxia (≥85% O_2_ for 36 h) compared with normoxic controls. **(C–E)** RT-qPCR analysis of ferroptosis-related genes acyl-CoA synthetase long-chain family member 4 (ACSL4), solute carrier family 7 member 11 (SLC7A11), and glutathione peroxidase 4 (GPX4). **(F)** Western blot analysis and quantification of YAP1 and ferroptosis-related proteins under different treatment conditions. Data are presented as mean ± SD from six independent biological replicates (n = 6). Statistical analysis was performed as described in the Materials and Methods. *p < 0.05, **p < 0.01, ***p < 0.001.

After confirming that hyperoxia induces a ferroptosis-like phenotype in AECs, we examined changes in the miR-21-5p/YAP1 axis and related ferroptosis-associated molecules. RT-qPCR analysis showed that hyperoFigures 3A,Bxia exposure decreased miR-21-5p expression while increasing YAP1 mRNA levels (Figures 4A,B). These findings suggest that the miR-21-5p/YAP1 axis may be dysregulated under hyperoxic conditions. Among ferroptosis-related markers, ACSL4 expression was increased in the hyperoxia-exposed group, whereas SLC7A11 and GPX4 were decreased (Figures 4C–E). Western blot results showed protein expression changes consistent with the mRNA data (Figure 4F). Treatment with the ferroptosis inhibitor Fer-1 partially reversed these molecular changes, supporting the involvement of ferroptosis in this process. In summary, hyperoxia exposure was associated with decreased miR-21-5p, increased YAP1, and altered expression of ACSL4, SLC7A11, and GPX4, changes consistent with ferroptosis-associated alterations.

### 3.5 miR-21-5p modulates hyperoxia-associated ferroptosis via YAP1

#### 3.5.1 Overexpression experiments

To investigate the functional role of the miR-21-5p/YAP1 axis in hyperoxia-induced ferroptosis, we performed miR-21-5p overexpression, YAP1 overexpression, and co-transfection rescue experiments in hyperoxia-treated AECs. The efficiency of YAP1 overexpression and knockdown was previously validated under normoxic conditions, and siRNA-702, which exhibited the highest knockdown efficiency, was selected for subsequent experiments (Supplementary Figure S1).

**FIGURE 5:**
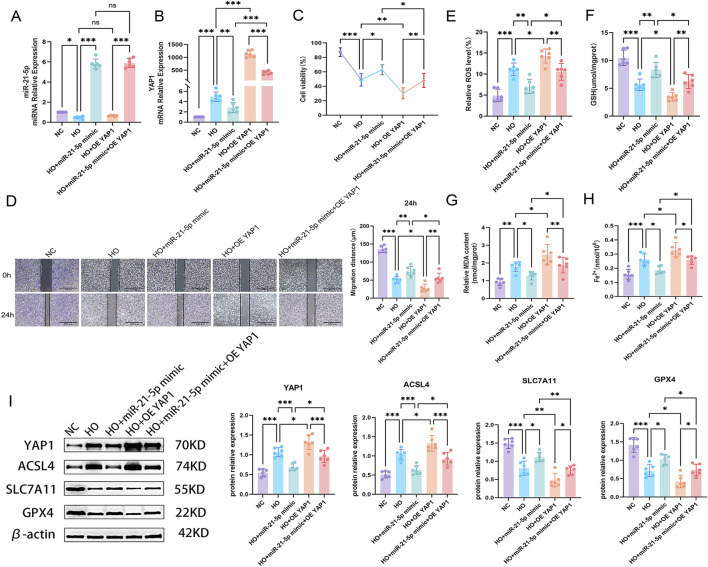
miR-21-5p overexpression alleviates hyperoxia-induced ferroptosis by targeting YAP1. **(A)** RT-qPCR analysis of miR-21-5p expression under hyperoxia in overexpression groups. **(B)** RT-qPCR analysis of YAP1 expression under hyperoxia. **(C)** Cell viability of AECs under hyperoxia in different transfection groups. **(D)** Cell migration assay and its quantitative analysis (scale bar = 500 μm). **(E–H)** Levels of ROS, GSH, MDA, and Fe^2+^. **(I)** Western blot analysis and quantification of YAP1 and ferroptosis-related proteins. Data are presented as mean ± SD from six independent biological replicates (n = 6). Statistical analysis was performed as described in the Materials and Methods. *p < 0.05, **p < 0.01, ***p < 0.001.

Under hyperoxic conditions, compared with the NC group, the HO group showed decreased miR-21-5p expression and increased YAP1 expression. miR-21-5p mimic increased miR-21-5p levels and reduced YAP1 expression, whereas YAP1 overexpression increased its mRNA levels. In the co-transfection group, miR-21-5p remained elevated, while YAP1 expression increased compared with the HO + miR-21-5p mimic group but remained lower than that in the YAP1 overexpression group (Figures 5A,B). These results indicate that the overexpression and rescue models were successfully established under hyperoxic conditions.

We next evaluated cellular functions and ferroptosis-related changes across the groups. CCK-8 assays showed that hyperoxia reduced cell viability, whereas miR-21-5p overexpression improved cell viability, and YAP1 overexpression further reduced cell viability. Under co-transfection conditions, miR-21-5p partially mitigated the effects of YAP1 overexpression (Figure 5C). Scratch assays showed a similar trend: miR-21-5p overexpression improved migration, whereas YAP1 overexpression reduced migration; under co-transfection conditions, miR-21-5p partially reversed the effects of YAP1 (Figure 5D). Regarding ferroptosis markers, hyperoxia led to increased ROS, MDA, and Fe^2+^ levels, accompanied by decreased GSH levels. miR-21-5p overexpression attenuated these changes, whereas YAP1 overexpression enhanced them. Under co-overexpression conditions, miR-21-5p partially reversed the effects induced by YAP1 (Figures 5E–H). Western blot analysis showed that miR-21-5p overexpression reduced YAP1 and ACSL4 expression, while increasing SLC7A11 and GPX4 levels; YAP1 overexpression showed opposite trends (Figure 5I), consistent with the observed cellular and ferroptosis-related changes.

#### 3.5.2 Inhibition experiments

**FIGURE 6:**
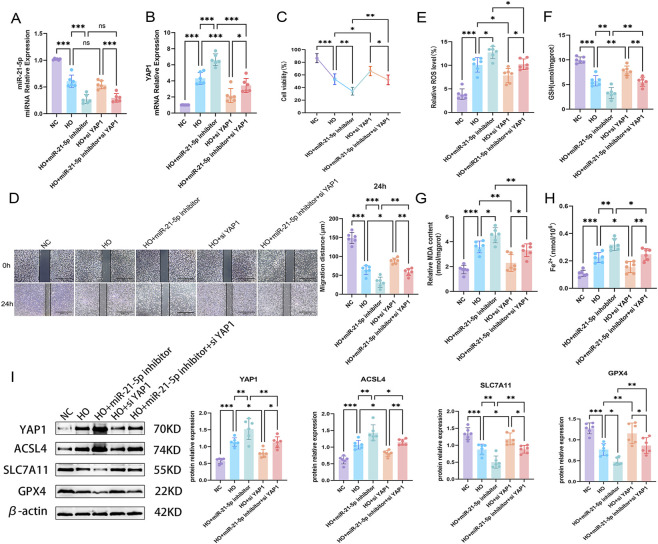
Inhibition of miR-21-5p aggravates ferroptosis, while YAP1 knockdown partially reverses this effect **(A)** RT-qPCR analysis of miR-21-5p expression under hyperoxia in inhibition groups. **(B)** RT-qPCR analysis of YAP1 expression under hyperoxia. **(C)** Cell viability of AECs under hyperoxia in different transfection groups. **(D)** Cell migration assay and its quantitative analysis (scale bar = 500 μm). **(E–H)** Levels of ROS, GSH, MDA, and Fe^2+^. **(I)** Western blot analysis and quantification of YAP1 and ferroptosis-related proteins. Data are presented as mean ± SD from six independent biological replicates (n = 6). Statistical analysis was performed as described in the Materials and Methods. *p < 0.05, **p < 0.01, ***p < 0.001.

RT-qPCR analysis showed that hyperoxia decreased miR-21-5p expression and increased YAP1 expression. miR-21-5p inhibition further decreased miR-21-5p levels and increased YAP1 expression, whereas siYAP1 did not significantly affect miR-21-5p expression. In the HO + inhibitor + siYAP1 group, miR-21-5p remained low. YAP1 expression was increased under HO conditions; miR-21-5p inhibition further increased YAP1 expression, whereas siYAP1 reduced YAP1 expression. In the HO + inhibitor + siYAP1 group, YAP1 levels were lower than in the HO + inhibitor group, indicating that the inhibition and rescue models were successfully established (Figures 6A,B).

Functional assays showed that miR-21-5p inhibition further aggravated hyperoxia-induced cellular injury, as reflected by reduced cell viability, whereas YAP1 knockdown partially restored viability (Figure 6C). Migration assays showed a similar trend (Figure 6D). Regarding ferroptosis-related markers, miR-21-5p inhibition increased ROS, MDA, and Fe^2+^ levels and decreased GSH levels, whereas siYAP1 partially reversed these changes (Figures 6E–H). Western blot analysis showed that miR-21-5p inhibition increased YAP1 and ACSL4 expression and decreased SLC7A11 and GPX4 levels, whereas siYAP1 partially reversed these changes (Figure 6I).

In summary, these findings suggest that miR-21-5p may modulate cellular antioxidant capacity in part through YAP1, thereby influencing hyperoxia-induced ferroptosis and lung injury.

### 3.6 Hyperoxia-induced lung injury and ferroptosis-related changes *In vivo*

**FIGURE 7:**
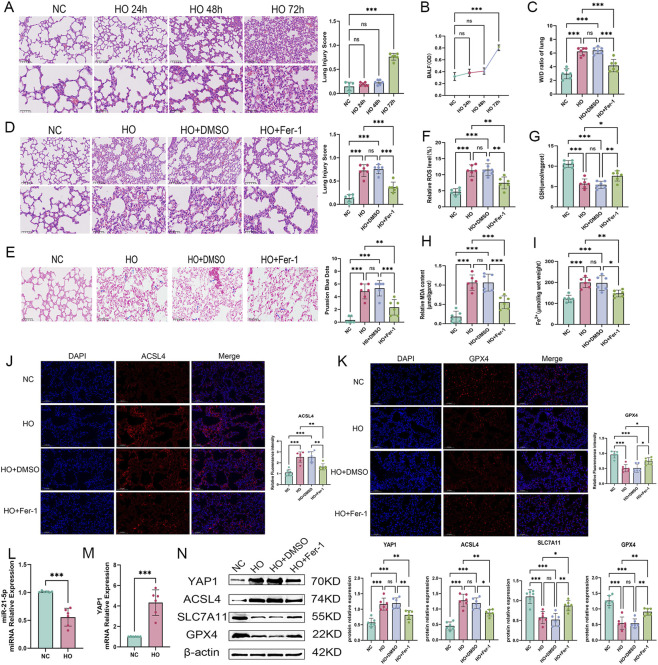
Phenotypic characterization and key gene validation in a HALI mouse model **(A)** Hematoxylin and eosin (HE) staining of mouse lung tissues after hyperoxia exposure at 0, 24, 48, and 72 h. Histopathological injury scoring was performed. Scale bars: 100 μm (top) and 50 μm (bottom). **(B)** Quantification of protein concentration in bronchoalveolar lavage fluid (BALF). **(C)** Lung wet-to-dry weight ratio (W/D). **(D)** HE staining of mouse lung tissues under different treatment conditions (HO, HO + DMSO, HO + Fer-1) and injury scoring. Scale bars: 100 μm (top) and 50 μm (bottom). **(E)** Prussian blue staining and quantification of iron deposition (scale bar = 50 μm). **(F–I)** ROS, GSH, MDA, and Fe^2+^ levels in lung tissues. **(J)** Immunofluorescence staining and quantification of ACSL4 (scale bar = 100 μm). **(K)** Immunofluorescence staining and quantification of GPX4 (scale bar = 100 μm). **(L–M)** RT-qPCR analysis of miR-21-5p and YAP1 expression. **(N)** Western blot analysis and quantification of YAP1 and ferroptosis-related proteins. Data are presented as mean ± SD (n = 6 mice per group). Statistical analysis was performed as described in the Materials and Methods. *p < 0.05,**p < 0.01,***p < 0.001).

To validate the *in vitro* findings *in vivo*, a murine HALI model was established. Mice were exposed to hyperoxia for 0, 24, 48, and 72 h to assess injury progression. Histological analysis revealed progressive alveolar wall disruption, septal thickening, and inflammatory infiltration, and lung injury scores showed a gradual increase (Figure 7A), accompanied by increased BALF protein levels, indicating enhanced alveolar–capillary permeability (Figure 7B). Accordingly, 72 h was selected for subsequent analyses.

Hyperoxia exposure resulted in increased pathological scores and wet-to-dry lung weight ratios, along with alveolar collapse and inflammatory infiltration (Figures 7C,D). Prussian blue staining revealed iron accumulation in lung tissue, which was partially reduced by Fer-1 treatment (Figure 7E). Biochemical assays showed increased ROS, MDA, and Fe^2+^ levels and decreased GSH content (Figures 7F–I). Immunofluorescence analysis demonstrated increased ACSL4 and reduced GPX4 expression following hyperoxia, both of which were partially restored by Fer-1 (Figures 7J,K). RT-qPCR and Western blot analyses further showed YAP1 upregulation and miR-21-5p downregulation in hyperoxia-exposed lungs (Figures 7L–N). In summary, these findings suggest that hyperoxia exposure is associated with dysregulation of the miR-21-5p/YAP1 axis and alterations in ferroptosis-related molecules, indicating that this axis may contribute to the development of HALI.

### 3.7 miR-21-5p deficiency aggravates hyperoxia-induced lung injury and ferroptosis-associated changes

**FIGURE 8:**
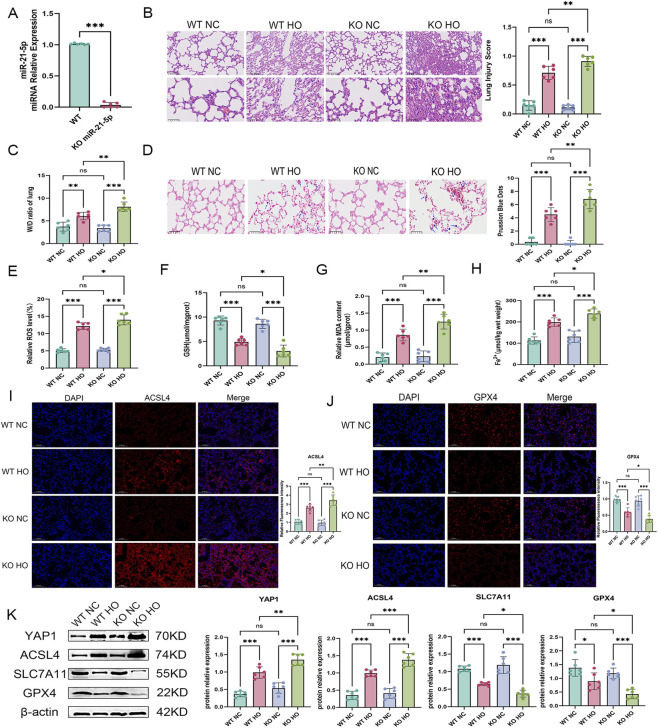
miR-21-5p deficiency exacerbates hyperoxia-induced lung injury and ferroptosis-related changes accompanied by increased YAP1 expression **(A)** RT-qPCR analysis of miR-21-5p expression in miR-21-5p knockout (KO) mouse lungs. **(B)** HE staining and injury scoring in wild-type (WT) and KO mice under control and hyperoxia conditions. Scale bars: 100 μm (top) and 50 μm (bottom). **(C)** Lung W/D ratio. **(D)** Prussian blue staining and quantification (scale bar = 50 μm). **(E–H)** ROS, GSH, MDA, and Fe^2+^ levels in lung tissues. **(I)** Immunofluorescence staining and quantification of ACSL4 (scale bar = 100 μm). **(J)** Immunofluorescence staining and quantification of GPX4 (scale bar = 100 μm). **(K)** Western blot analysis and quantification of YAP1 and ferroptosis-related proteins. Data are presented as mean ± SD (n = 6 mice per group). Statistical analysis was performed as described in the Materials and Methods. *p < 0.05,**p < 0.01,***p < 0.001.

To further investigate the role of miR-21-5p *in vivo*, HALI was induced in miR-21-5p knockout (KO) mice. RT-qPCR analysis confirmed near-complete loss of miR-21-5p expression in KO lung tissues (Figure 8A). Compared with WT mice, KO animals exhibited more severe alveolar damage, higher pathological scores, and increased wet-to-dry lung weight ratios (W/D) following hyperoxia exposure (Figures 8B,C).Prussian blue staining revealed increased iron accumulation in KO lung tissues (Figure 8D). Biochemical assays showed increased ROS, MDA, and Fe^2+^ levels, along with reduced GSH content in KO mice (Figures 8E–H). Immunofluorescence and Western blot analyses showed increased YAP1 and ACSL4 expression and decreased SLC7A11 and GPX4 expression in KO lung tissues (Figures 8I–K).

Together, these findings suggest that miR-21-5p deficiency is associated with enhanced ferroptosis-related alterations and aggravated lung injury under hyperoxic conditions.

## 4 Discussion

**FIGURE 9:**
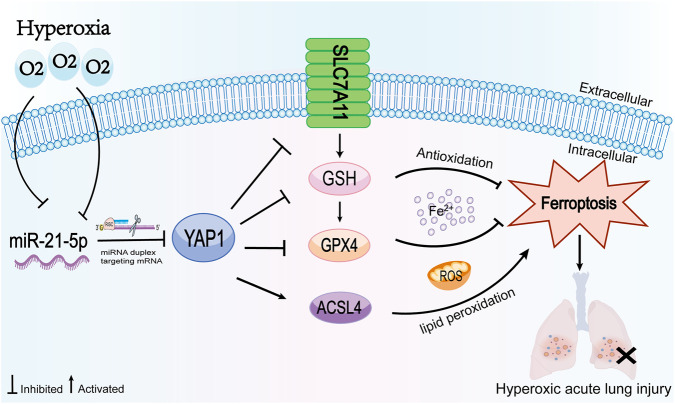
Schematic illustration of a proposed mechanism by which miR-21-5p is associated with ferroptosis-related changes through YAP1 in HALI. Hyperoxia exposure is associated with reduced miR-21-5p expression and increased YAP1 expression. These changes are accompanied by increased ACSL4 expression and decreased SLC7A11 and GPX4 expression, together with enhanced lipid peroxidation and impaired antioxidant capacity, ultimately contributing to ferroptosis-related lung injury.

This study systematically identifies a previously underrecognized regulatory axis—the miR-21-5p–YAP1–ferroptosis pathway—based on comprehensive *in vivo* and *in vitro* evidence. We demonstrate that hyperoxia markedly suppresses miR-21-5p expression, thereby releasing its inhibitory effect on YAP1 and resulting in aberrant YAP1 activation. Elevated YAP1 activity reshapes the lipid metabolic–antioxidant balance by enhancing ACSL4-associated lipid peroxidation while concurrently impairing the SLC7A11/GPX4-dependent antioxidant defense system. As a consequence, AECs are driven beyond the ferroptosis threshold (Figure 9). Restoration of miR-21-5p expression or inhibition of YAP1 partially re-establishes iron homeostasis and redox balance, effectively mitigating HALI. Collectively, these findings highlight ferroptosis as a pivotal pathological mechanism in HALI and introduce a novel upstream miRNA–transcriptional co-activator regulatory network governing ferroptotic susceptibility.

Although hyperoxic therapy remains indispensable in critical care, prolonged or excessive oxygen exposure disrupts the alveolar–capillary barrier, promotes HALI, and accelerates the progression of ARDS and BPD. Previous studies have shown that hyperoxia induces pronounced oxidative stress responses in alveolar epithelial and capillary endothelial cells, manifested by excessive ROS generation, lipid peroxidation, and dysregulated inflammatory mediator release, thereby forming a vicious cycle of “oxidative stress–membrane damage–inflammatory cascade” (Zhou et al., 2025). In recent years, multiple acute lung injury models have been reported to exhibit hallmark features of ferroptosis, including Fe^2+^ accumulation, lipid peroxide overload, reduced GPX4 activity, and GSH depletion (Stockwell, 2022). Jia et al. demonstrated in neonatal rat models that hyperoxia exposure significantly increased Fe^2+^ and MDA levels while reducing GPX4 activity, and that the ferroptosis inhibitor Fer-1 partially alleviated lung injury (Jia et al., 2021). Additional studies have shown that small molecules such as salidroside, wedelolactone, and urolithin A attenuate lung injury in part through suppression of ferroptosis-related pathways (Guo L. et al., 2022; Lou et al., 2023; Li et al., 2024). Collectively, these findings suggest that ferroptosis represents an important pathological process contributing to the progression of hyperoxia-induced lung injury rather than a mere secondary consequence.

Consistent with these observations, the present study further demonstrates ferroptosis-associated alterations in both *in vivo* and *in vitro* hyperoxia models. In hyperoxia-exposed AECs and mouse lung tissues, increased ROS, MDA, and Fe^2+^ levels, reduced GSH content, and prominent iron deposition were observed, accompanied by functional and structural lung impairment. Treatment with Fer-1 partially reversed these biochemical and pathological changes. These findings extend the conventional pathological framework of HALI beyond oxidative stress–mediated membrane damage and alveolar barrier disruption to include ferroptosis associated with dysregulated iron and redox homeostasis. Given the emerging importance of ferroptosis in HALI, this study further focused on miRNA-mediated post-transcriptional regulation to explore upstream mechanisms influencing ferroptotic susceptibility.

Among candidate miRNAs, miR-21-5p has been consistently reported to exert protective effects in multiple lung injury models. Qin et al. showed that hyperoxic exposure is associated with marked downregulation of miR-21-5p in alveolar type II epithelial cells, and that miR-21-5p modulates apoptosis through PTEN/Akt and STAT3 signaling pathways (Qin et al., 2019; 2023). The transcription factor Gata3 has been identified as an upstream regulator that promotes miR-21-5p transcription, thereby attenuating hyperoxia-induced lung injury (Ren et al., 2025). In addition, miR-21-5p enriched in AEC II-derived exosomes suppresses PGAM5-mediated necroptosis and mitigates hyperoxic lung injury (Yuan et al., 2025). However, previous studies have largely focused on classical forms of regulated cell death, including apoptosis, necroptosis, and autophagy, whereas systematic evidence regarding the involvement of miR-21-5p in ferroptosis regulation has remained limited.

Building upon these findings, the present study demonstrates that miR-21-5p not only modulates apoptotic pathways but also influences ferroptosis-associated processes. In both *in vivo* and *in vitro* models, miR-21-5p overexpression and genetic deletion were associated with attenuation and enhancement of ferroptosis-related phenotypes, respectively. These observations support a role for miR-21-5p as an upstream regulatory factor influencing susceptibility to multiple forms of regulated cell death under hyperoxic stress.

At the execution level of ferroptosis, ACSL4, SLC7A11, and GPX4 constitute a metabolic–antioxidant regulatory module that determines cellular vulnerability to lipid peroxidation. ACSL4 promotes polyunsaturated fatty acid incorporation into membrane phospholipids, thereby increasing the availability of oxidizable lipid substrates (Doll et al., 2017). In contrast, SLC7A11 functions as the rate-limiting component of the cystine/glutamate antiporter system Xc- and supports glutathione synthesis, while GPX4 catalyzes the reduction of phospholipid hydroperoxides and limits lipid peroxidation (Koppula et al., 2021; Li et al., 2020; Stockwell et al., 2017; Ingold et al., 2018). When ACSL4-driven lipid substrate accumulation coincides with impaired SLC7A11 and GPX4 activity, cells become increasingly susceptible to ferroptosis-associated lipid damage.

Our data indicate that hyperoxia-associated ferroptosis reflects an imbalance between lipid metabolic demand and antioxidant defense capacity. The miR-21-5p/YAP1 axis appears to modulate this balance by reshaping the ACSL4–SLC7A11–GPX4 network. Downregulation of miR-21-5p under hyperoxic stress is associated with increased YAP1 activity, which correlates with elevated ACSL4 expression and reduced SLC7A11 and GPX4 levels. Conversely, restoration of miR-21-5p expression or YAP1 suppression partially reverses these molecular alterations and attenuates ferroptosis-associated phenotypes in HALI.

As a core transcriptional coactivator in the Hippo pathway, YAP1 plays crucial roles in lung development, alveolar regeneration, and post-inflammatory repair. Previous studies suggest that in some ALI or sepsis models, YAP1 exerts an “anti-ferroptotic” effect by inhibiting ferritinophagy-mediated ferroptosis (Zhang et al., 2022), reducing ferritin degradation, and suppressing free iron release; during lung injury repair, Yap/Taz activation is also considered to aid alveolar epithelial regeneration (LaCanna et al., 2019). Furthermore, in an ALI model associated with intestinal ischemia-reperfusion, YAP exhibits lung protective effects by inhibiting ferroptosis through activation of the Nrf2/GPX4/SLC7A11 axis (Tang et al., 2025). In contrast, our findings indicate that under hyperoxic stress, increased YAP1 activity is associated with enhanced ACSL4 expression and impaired SLC7A11/GPX4 antioxidant capacity, thereby favoring ferroptosis-related injury in AECs. These differences likely reflect context-dependent regulatory outputs of YAP1 signaling shaped by distinct upstream cues and cellular environments, rather than contradictory intrinsic functions of YAP1.

Notably, our data revealed an asymmetric relationship between miR-21-5p and YAP1 under hyperoxic conditions: inhibition of miR-21-5p increased YAP1 expression, whereas inhibition of YAP1 did not significantly alter miR-21-5p levels. This nonreciprocal pattern suggests that the miR-21-5p/YAP1 interaction is organized predominantly as a unidirectional regulatory axis, with miR-21-5p functioning upstream of YAP1 rather than forming a classical feedback loop. Under hyperoxic stress, downregulation of miR-21-5p may therefore represent an early event that releases YAP1 from inhibitory control, thereby promoting ferroptosis-related metabolic and redox imbalance. This asymmetry also supports the view that miR-21-5p acts as an upstream regulator of ferroptotic susceptibility, whereas YAP1 functions mainly as a downstream effector that amplifies the injury program. From a translational perspective, restoration of miR-21-5p may therefore provide broader regulatory benefit than targeting YAP1 alone. However, indirect feedback from YAP1 to miR-21-5p cannot be completely excluded in other cellular contexts, at different stages of injury, or over longer time scales. In this regard, the biological output of YAP1 signaling is likely to be context dependent rather than fixed. YAP1 may thus exert divergent regulatory effects across pathological settings, promoting tissue repair in some inflammatory conditions while contributing to ferroptosis susceptibility under severe oxidative stress. These observations highlight the importance of considering microenvironmental context when targeting YAP1 signaling in lung injury.

This study has several limitations. First, the public transcriptomic datasets used in this study were primarily applied for candidate gene screening and hypothesis generation, rather than for formal cross-dataset integrative analysis. Therefore, these results should be interpreted as supportive evidence for target prioritization, while the main conclusions of this study rely on subsequent *in vitro* and *in vivo* validation. Second, although the biological replicates in the present study have been increased to strengthen the robustness of the findings, the overall sample size remains modest. In addition, RNA and protein analyses were mainly performed on adherent cells after treatment, which may not fully represent the entire cell population, particularly detached or dead cells. Although negative and vehicle controls were included, dedicated toxicity titration experiments for each transfection reagent/vector were not performed, and minor nonspecific effects therefore cannot be completely excluded. Moreover, in co-transfection experiments, differences in transfection efficiency between miRNA mimics and plasmid constructs may result in unequal intracellular expression of the introduced components, thereby potentially influencing the observed regulatory effects. These factors may affect the robustness and generalizability of the findings and warrant further validation in larger sample sizes. Third, although previous studies have suggested that YAP1 may regulate ACSL4, SLC7A11, and GPX4 (Lv et al., 2024), direct evidence at the chromatin level (e.g., ChIP-based assays) is lacking in this study, and the regulatory mechanism remains to be further clarified. Fourth, this study was conducted mainly in murine models, and validation in human-derived cells or organoids is needed to assess translational relevance. In addition, analyses based on whole lung tissue do not exclude contributions from other cell types. Finally, although Fer-1 was used as a ferroptosis inhibitor, additional pharmacological or genetic approaches will be needed to further substantiate pathway specificity.

Overall, this study reinforces the role of ferroptosis as an important contributor to hyperoxia-associated lung injury and integrates a miRNA–transcriptional co-activator regulatory axis into its upstream signaling framework. By characterizing the miR-21-5p/YAP1/ACSL4–SLC7A11–GPX4 regulatory network, this work refines the mechanistic understanding of HALI pathogenesis and provides a conceptual basis for future studies targeting ferroptosis modulation under hyperoxic stress.

## 5 Conclusion

In summary, using complementary *in vivo* and *in vitro* models, this study characterizes a regulatory role for the miR-21-5p/YAP1 axis in hyperoxia-associated ferroptosis. Hyperoxic exposure is associated with miR-21-5p downregulation and increased YAP1 activity, accompanied by altered expression of ACSL4, SLC7A11, and GPX4, thereby favoring lipid peroxidation and ferroptosis-related lung injury. Restoration of miR-21-5p or inhibition of YAP1 partially rebalances this antioxidant network and attenuates ferroptosis-associated damage.

By extending prior work that primarily focused on apoptosis and autophagy, this study highlights ferroptosis as an important regulated cell death pathway in hyperoxia-induced lung injury and identifies a miRNA-mediated signaling axis that modulates ferroptosis execution. These findings provide mechanistic insight into HALI pathogenesis and support further investigation of miR-21-5p/YAP1 signaling as a potential target for limiting ferroptosis under hyperoxic conditions.

## Data Availability

The original contributions presented in the study are included in the article/Supplementary Material, further inquiries can be directed to the corresponding author.
