# Supplementary material for: miR-21-5p attenuates hyperoxia-induced lung injury by modulating YAP1-dependent ferroptosis
Source: Front Pharmacol. 2026 May 11;17:1804152. doi: 10.3389/fphar.2026.1804152 (PMC13199173; doi:10.3389/fphar.2026.1804152)
Supplement: Supplementary file 1 [file Table1.docx]

**Supplementary Table S1**. Sequences of oligonucleotides and plasmid constructs used in this study

| Name | Type | Sequences/Backbone |
| --- | --- | --- |
| miR-21-5p mimic | mimic | F UAGCUUAUCAGACUGAUGUUGA  R AACAUCAGUCUGAUAAGCUAUU |
| miR-21-5p mimic NC | negative control | F UUCUCCGAACGUGUCACGUTT  R ACGUGACACGUUCGGAGAATT |
| miR-21-5p inhibitor | inhibitor | UCAACAUCAGUCUGAUAAGCUA |
| miR-21-5p inhibitor NC | negative control | CAGUACUUUUGUGUAGUACAA |
| OE YAP1 | plasmid | Full-length mouse YAP1 coding sequence cloned into pEX-3; empty pEX-3 vector used as negative control (OE NC)  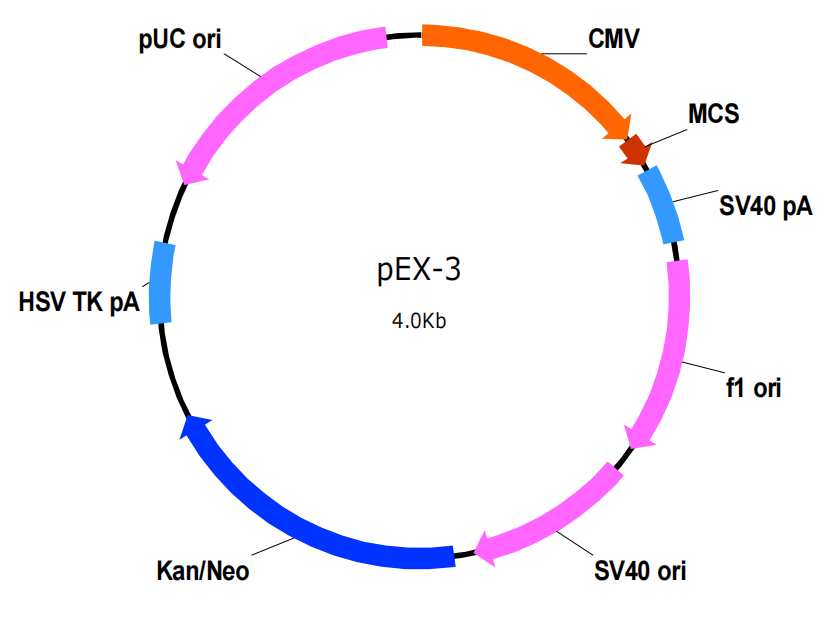 |
| YAP1-Mus-1116 | siRNA | F GUUGAAACAACAGGAAUUATT  R GUUGAAACAACAGGAAUUATT |
| YAP1-Mus-1225 | siRNA | F CAGGAAUUGAGAACAAUGATT  R UCAUUGUUCUCAAUUCCUGTT |
| YAP1-Mus-702 | siRNA | F GACAUCUUCUGGUCAAAGATT  R UCUUUGACCAGAAGAUGUCTT |
| YAP1-Mus-828 | siRNA | F GCUGAUGAAUUCUGCCUCATT  R UGAGGCAGAAUUCAUCAGCTT |
| YAP1 WT-miR-21-5p | luciferase reporter (WT) | GAACAAAATAGGCATGAATTAACTCTGTGTATAAGCTATGAAGTAATAGTTGGTTATGAATTATAGT |
| YAP1 MUT-miR-21-5p | luciferase reporter (MUT) | GAACAAAATAGGCATGAATTAACTCTGTGTTATTCGAATGAAGTAATAGTTGGTTATGAATTATAGT |
