# Supplementary figures and images for: miR-21-5p attenuates hyperoxia-induced lung injury by modulating YAP1-dependent ferroptosis
Source: Front Pharmacol. 2026 May 11;17:1804152. doi: 10.3389/fphar.2026.1804152 (PMC13199173; doi:10.3389/fphar.2026.1804152)

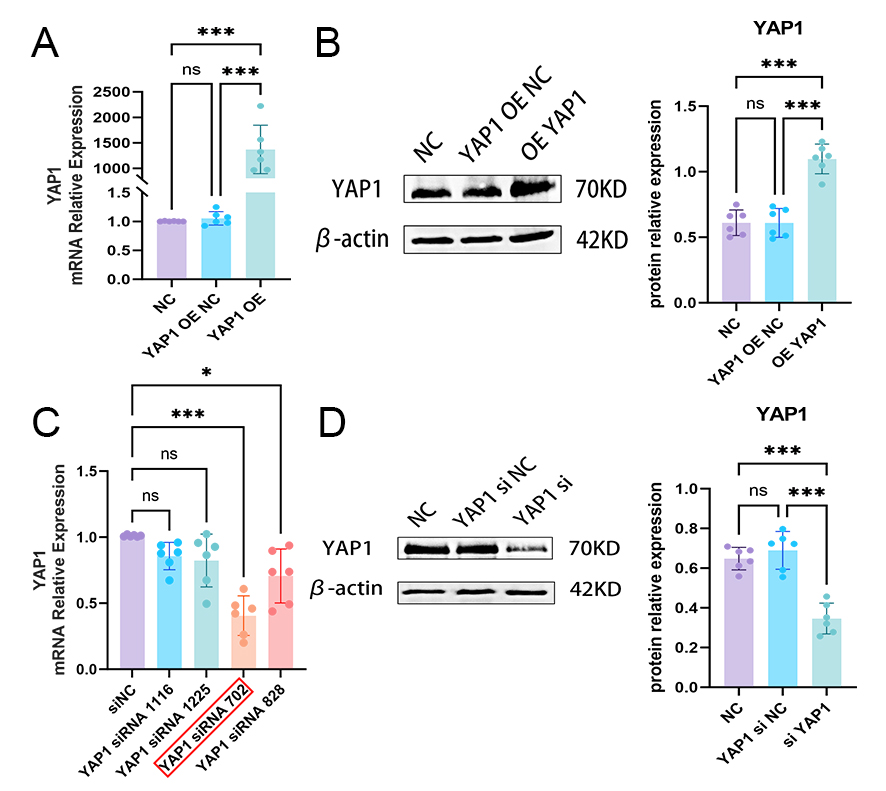

Supplement: Supplementary file 2 [file Image1.jpeg]
